# The Potential Application of Nanocarriers in Delivering Topical Antioxidants

**DOI:** 10.3390/ph18010056

**Published:** 2025-01-06

**Authors:** Zulfan Zazuli, Rika Hartati, Cornelia Rosasepti Rowa, Sukmadjaja Asyarie, Satrialdi

**Affiliations:** 1 Department of Pharmacology and Clinical Pharmacy, School of Pharmacy, Institut Teknologi Bandung, Bandung 40132, Indonesia; zulfan@itb.ac.id; 2 Department of Pharmaceutical Biology, School of Pharmacy, Institut Teknologi Bandung, Bandung 40132, Indonesia; rikahar@itb.ac.id; 3 Department of Pharmaceutics, School of Pharmacy, Institut Teknologi Bandung, Bandung 40132, Indonesia; corneliarowa@gmail.com (C.R.R.); asyariesukmadjaja@gmail.com (S.A.)

**Keywords:** nanocarriers, antioxidants, topical, natural antioxidants

## Abstract

The imbalance in the production of reactive oxygen species (ROS) with endogenous antioxidant capacity leads to oxidative stress, which drives many disorders, especially in the skin. In such conditions, supplementing exogenous antioxidants may help the body prevent the negative effect of ROS. However, the skin, as the outermost barrier of the body, provides a perfect barricade, making the antioxidant delivery complicated. Several strategies have been developed to enhance the penetration of antioxidants through the skin, one of which is nanotechnology. This review focuses on utilizing several nanocarrier systems, including nanoemulsions, liposomes, solid lipid nanoparticles (SLNs), nanostructured lipid carriers (NLCs), and polymeric nanoparticles, for transporting antioxidants into the skin. We also reveal ROS formation in the skin and the role of antioxidant therapy, as well as the natural sources of antioxidants. Furthermore, we discuss the clinical application of topical antioxidant therapy concomitantly with the current status of using nanotechnology to deliver topical antioxidants. This review will accelerate the advancement of topical antioxidant therapy.

## 1. Introduction

**Figure 1:**
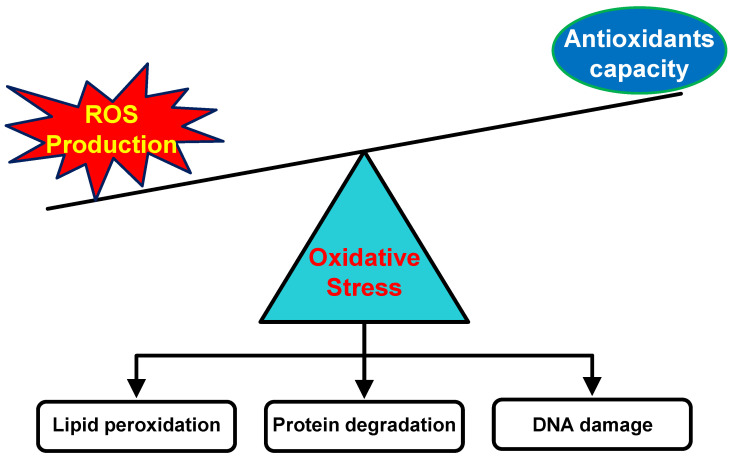
The disproportion in ROS production and endogenous antioxidant capacity leads to oxidative stress that may induce various pathological conditions.

As the outermost organ, the skin is vital as the first barrier for protecting the body from hazardous materials, pathogenic invasion, and mechanical injury. Maintaining the skin in a healthy condition is very important to ensure its role can be adequately functional. In addition, skin quality also plays an essential part in human attractiveness. The intensive exposure to external factors such as ultraviolet (UV) radiation, air pollution, and some xenobiotics causes excessive free radicals in the skin. Free radicals are unstable and very reactive molecular species that contain one or more unpaired electrons on their orbital atom [1]. One of the most prominent and biologically relevant free radicals that are naturally produced by the cells during the metabolic process is oxygen-derived free radicals, known as reactive oxygen species (ROS). Under normal conditions, ROS involve various signaling pathways for regulating several physiological and biological processes. However, the uncontrolled production of ROS may damage cell components and functional cellular molecules [2,3], due to the highly reactivity properties of ROS against biomolecules. Naturally, the skin is equipped with an antioxidant system to control and maintain ROS homeostasis [4]. The imbalance in the production of free radicals and the neutralizing capacity of the endogenous antioxidant system leads to oxidative stress (Figure 1), which can induce several pathological conditions. In this condition, the supplementation of exogenous antioxidants may help reduce and control the negative impact of the oxidative stress.

Topical administration of antioxidants contributes to strengthening the endogenous antioxidant capacity to prevent the adverse effects of increasing the amount of ROS in the skin [5,6]. Moreover, the topical route provides a localized and targeted effect on the skin, minimizes the systemic adverse effects, and prevents the first-pass metabolism following oral administration [7]. Various conventional topical dosage forms are widely used for delivering drugs, including ointment, cream, lotion, topical solution, topical suspension, gel, foam, and spray. However, the existence of a stratum corneum on the utmost part of the skin poses enormous challenges in delivering antioxidants, mainly using conventional topical dosage forms. Moreover, the solubility and stability issues of some antioxidant agents are still obstacles to developing optimal therapeutic strategies. Therefore, the development of a novel drug delivery system is very relevant to improve antioxidant therapy efficacy.

The invention of drug delivery antioxidant therapy aligns with market needs from a business perspective. The global market for cosmetic antioxidants is experiencing growth throughout time. In 2023, it was valued at USD 145.20 million, and is projected to attain around USD 253.24 million by 2033, with a compound annual growth rate (CAGR) of 5.72% from 2024 to 2033 [8]. A separate source indicates that the worldwide antioxidants market was valued at USD 4.59 billion in 2023 and is projected to increase from USD 4.84 billion in 2024 to USD 7.64 billion by 2032, reflecting a CAGR of 5.87% throughout the forecast period [9]. Both sources concur that the Asia Pacific region dominates the antioxidants market, holding a market share of 43.79% in 2023.

Back in 1959, the Nobel Prize laureate Richard Feynman introduced the concept of small-scale things, which later developed into nanotechnology [10]. This technology attracts much attention, especially in the medical field, since it has several advantages due to its nanoscale size that can be used for imaging, diagnosis, and drug delivery. In dermatological therapies, nanotechnology has made revolutionary advancements by overcoming traditional barriers such as poor penetration of therapeutic agents through the skin’s outer layer, the stratum corneum [11,12]. An important development in this field is the use of antioxidants encapsulated in nanocarriers to combat oxidative stress—a key contributor to skin aging, inflammation, and many dermatological conditions [13,14]. Researchers have enhanced the bioavailability and efficacy of antioxidants by stabilizing them within nanocarriers. These nanoformulations protect antioxidants from degradation due to environmental factors [13,14]. Consequently, nanocarriers infused with antioxidants have become essential in antiaging products and treatments designed to mitigate oxidative damage and enhance skin health.

This review focuses on the potential application of the nanocarrier system in delivering antioxidants through topical administration. We summarize five types of nanocarrier systems that can be implemented as topical antioxidant carriers, including nanoemulsions, liposomes, solid lipid nanoparticles (SLNs), nanostructured lipid carriers (NLCs), and polymeric nanoparticles. Moreover, the origin of ROS in the skin and the application of topical antioxidant therapy are revealed. This review also summarizes the potential of natural resources as the source of antioxidants. Additionally, we delve into the practical use of topical antioxidant therapy in clinical settings and explore the latest developments in leveraging nanotechnology to deliver topical antioxidants.

## 2. Overview of the Skin Structure and the Related Challenges in Topical Antioxidant Delivery

The skin is the largest and outermost organ of the human body. It serves several essential functions, including acting as a mechanical barrier to protect against external materials, pathogens, and UV radiation. The skin also helps regulate the body temperature, prevents excessive loss of endogenous substances, and contains sensory organs. Additionally, it plays a role in vitamin D production and is an important factor in human attractiveness. The integumentary system comprises three primary layers: the epidermis, the dermis, and the hypodermis. The epidermis is further categorized into two distinct sections: the nonviable stratum corneum and the viable layers, which include the stratum lucidum, stratum granulosum, stratum spinosum, and stratum basale. The dermis represents the most substantial component of the skin, housing numerous essential structures such as sebaceous glands, sweat glands, hair follicles, blood vessels, and sensory nerves. The hypodermis, also known as the subcutaneous layer, is the innermost layer of the skin, made up of adipose (fat) and connective tissues that primarily function in terms of shock absorption and insulation. The complex architecture of the skin presents significant challenges for the drug delivery process.

As the most superficial part of the integumentary system, the stratum corneum is well known as the most relevant barrier in terms of the drug delivery process. The stratum corneum is made up of corneocytes, which are anucleate keratinocytes. These cells are filled with hydrophilic keratin proteins and are tightly surrounded by hydrophobic lipid lamellae, which are composed of cholesterol, free fatty acids, and ceramides [15]. Furthermore, the stratum corneum, along with tight junctions and desmosomes, creates a significant mechanical and permeability barrier. This barrier prevents endogenous materials from escaping the skin while also being a strong defense against exogenous materials entering the skin [16]. The penetration of drugs into the skin can be facilitated through three distinct mechanisms: the intercellular route, the transcellular route, and the appendageal route [17]. The intercellular route allows for drug permeation through the spaces between the corneocytes in the stratum corneum. In contrast, the transcellular route involves the passage of the drug through the lipid lamellae and the corneocytes. Additionally, the appendageal route permits the movement of polar and relatively large molecules by exploiting the openings created by hair follicles, sweat glands, and sebaceous glands within the skin. However, it is important to note that the skin area characterized by these openings is minimal, limiting the effectiveness of the appendageal route. In 2000, Bos and Meinardi established the 500 Dalton rule, which states that only small molecules with a cutoff size of 500 Daltons and lipophilic characteristics can penetrate the skin, while macromolecules are unable to do so [18]. In conjunction with these facts, several strategies have been developed to improve the skin penetration abilities of the drugs.

At least three primary mechanisms have been developed to enhance drug penetration through the skin: chemically enhanced methods, physically enhanced methods, and stimuli-enhanced methods [19]. Chemically enhanced methods utilize chemical compounds, such as skin penetration enhancers and occlusive materials, to improve the permeability of the skin. In contrast, physically enhanced methods utilize techniques that bypass the stratum corneum, such as ablation procedures to remove this outer layer or the application of microneedles, which allow for direct drug delivery into the deeper layers of the skin. Additionally, external stimuli such as thermal energy, electrical currents, and ultrasound can be used to enhance the transfer of drugs through the stratum corneum, namely the stimuli-enhanced methods.

The methods mentioned above are not without several drawbacks, ranging from the lack of effectiveness of penetration into the skin and the need for a professional to conduct the protocol to several side effects such as irritation, discomfort, and pain [19]. Consequently, drug delivery to the skin continues to be an active area of research. One emerging strategy is the use of nanotechnology in the process of drug delivery to the skin. The unique characteristics of nanomaterials can be exploited to maximize drug penetration into the skin as well as the protection of the cargo from being degraded while minimizing the shortcomings of other delivery strategies.

## 3. The Origin of ROS in the Skin

**Figure 2:**
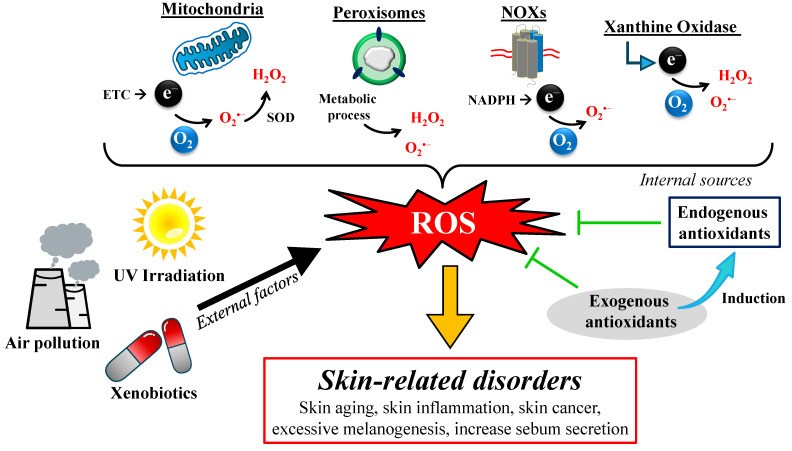
Various metabolic processes and external factors can increase ROS production in the skin. While endogenous antioxidants help maintain redox homeostasis, an imbalance between ROS and antioxidant capacity can lead to skin disorders. Supplementing exogenous antioxidants can support the body’s antioxidant defenses by scavenging ROS and boosting endogenous antioxidant activity.

ROS is the natural by-product of metabolism, mainly produced during the electron transport chain (ETC) process in the mitochondria (Figure 2). ETC is a critical step in energy synthesis, in which the electrons are transferred through several complex proteins located on the inner mitochondrial membrane. The leakage of electrons from the complex I and complex III of the ETC leads to a partial reduction in the oxygen molecules to form a highly reactive radical species, namely superoxide (O_2_^•−^) [20]. In the presence of SOD, superoxide undergoes dismutation to form another ROS, namely hydrogen peroxide (H_2_O_2_). H_2_O_2_ has relatively stable characteristics and is membrane-permeable; thus, it can be transferred into the cytosol [21]. The dysfunction of the ETC may lead to a higher production of ROS. For example, rotenone and piericidin interrupt the electron transport to CoQ10, enhancing ROS production in the mitochondria [22].

ROS are also produced as a by-product of several enzymatic processes (Figure 2). NADPH oxidases (NOXs), membrane-spanning enzymes, are responsible for generating ROS. Electrons are transferred from cytosolic NADPH to oxygen molecules on the opposite side of the cell membrane via FAD and heme, generating superoxide by NOX1-3 or NOX5, or H_2_O_2_ by NOX4 or DUOX1-2 [23]. Another enzyme responsible for inducing ROS generation is a xanthine oxidase (XO), which catalyzes the oxidative hydroxylation of purine molecules, producing superoxide and H_2_O_2_ by-products [24]. According to Higa et al., inhibiting XO with febuxostat significantly suppresses ROS generation [25], suggesting the close connection between XO activity and the production of ROS. The cell also generates ROS during metabolic processes in peroxisomes and xenobiotic metabolism via cytochrome P450 enzymes.

Several exogenous factors also play a critical role in the induction of ROS generation, including UV irradiation, exposure to pollution, and several xenobiotics (Figure 2). The prolonged contact with UV irradiation may directly induce the deoxyribonucleic acid (DNA) mutation through DNA strand breaks (DSBs), leading to the failure of functional protein synthesis [26]. It is well known that nuclear and mitochondrial DNA encodes the proteins responsible for cellular respiration. The mutation of the DNA may directly induce dysfunction in the cellular respiration process, accelerating ROS production. There are at least two ways that UV irradiation can cause the generation of ROS in cells [27]. The first mechanism is through direct light absorption by the cell and its components, leading to subsequent photochemical reactions. The second mechanism is via photosensitization, which is mediated by several endogenous photosensitizers like flavins, bilirubin, melanin, porphyrins, and other photoactivated molecules [28]. The absorbed UV light excites the sensitizers into their triplet state, followed by type I or type II photochemical reactions, resulting in the production of free radicals or singlet oxygen, respectively.

Pollution, such as polycyclic aromatic hydrocarbons (PAHs), volatile organic compounds (VOCs), and particulate matter (PM) are potential sources of exposure. PAHs are mostly caused by the smoke produced when organic matter like wood or oil is burned, which can generate ROS and stimulate the growth of melanocytes. When exposed to UV radiation, they stimulate melanin production, leading to the creation of sun-induced lentigines and increasing the risk of skin cancer (papilloma, carcinoma, and squamous cell carcinoma). The food is a primary source of PAH contamination, primarily from consuming smoked meat [29,30].

Various effects of exposure to volatile organic compounds (VOCs) have become an important issue for human health due to their widespread use in industries. A study reported that malondialdehyde (MDA) and SOD were significantly higher in textile workers than in controls [31]. Elevated MDA levels in textile workers may indicate increased lipid peroxidation as a result of long-term exposure to organic solvents, whereas elevated SOD activity suggests that the antioxidant system was activated to counter lipid peroxidation. A study from Saudi conducted on paint workers showed similar results [32]. Another study reported that the indication of oxidative stress induced by VOC exposure may have a significant impact on childhood asthma [33]. However, few studies have focused on the impact of VOCs on the typical physiological processes that occur in the skin.

The primary air pollutant is PM, typically emitted by factories, power plants, incinerators, vehicles, the construction sector, and fires. However, its properties vary depending on the location of the emissions (rural, urban, or industrial areas) and the sampling site [6]. Ultrafine particles, thin particles, and PAHs can interact more effectively with the skin due to their size, leading to toxic effects such as pro-oxidant, mutagenic, and carcinogenic activity. In some cases, these substances can react with the sunlight, causing phototoxicity [29,34]. Contaminants can enter the human body by penetrating the stratum corneum or entering the systemic circulation. Transcutaneous penetration is the process by which particles travel through the sebum and many layers of the stratum to reach the first live keratinocytes [35].

## 4. The Role of Antioxidant Therapy in Minimizing Harmful Effects of Oxidative Stress

**Figure 3:**
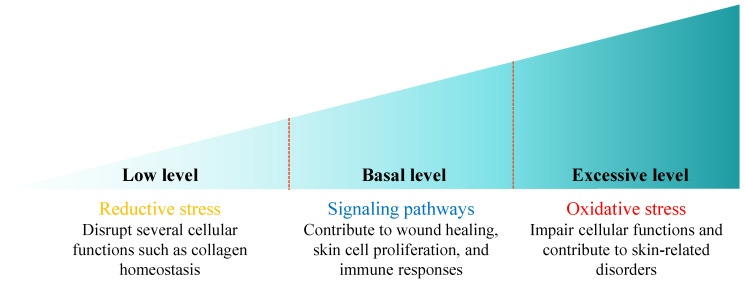
ROS play different roles in the skin depending on their concentration levels. At low concentrations, ROS can lead to reductive stress, which negatively impacts various cellular functions. While ROS are beneficial for the skin at the basal levels, excessive amounts can impair these functions and result in various skin disorders.

ROS serve as a double-edged sword within cellular processes, with their effects largely dependent on their concentration in the cells (Figure 3). At the basal concentrations, ROS play a vital role as signaling molecules beneficial to the skin. They support various physiological pathways, particularly those associated with wound healing [36], enhance skin cell proliferation [37], and modulate immune responses to ensure an effective defense mechanism [38]. Conversely, when ROS are produced in excessive amounts, they can become harmful. Overproduction of ROS leads to oxidative stress, which can disrupt the integrity and function of critical biomolecules, such as lipids, DNA, and proteins [2,3]. This oxidative damage may impair cellular functions and contribute to various skin-related conditions. The opposite condition can occur when ROS production is extremely low, leading to reductive stress that may disrupt various cellular functions, one of which disrupts collagen homeostasis in human dermal fibroblasts [39]. Thus, understanding the balance of ROS concentration is essential for utilizing their beneficial properties while mitigating their potentially harmful effects.

To minimize the harmful effects of ROS, the skin is equipped with several natural defense mechanisms, including an antioxidant system such as superoxide dismutase (SOD), catalase (CAT), glutathione peroxidase (GPx), glutathione reductase (GR), peroxiredoxins (Prxs) [4], and biofunctional molecules. However, in some cases, the generation of ROS is uncontrolled, so that oxidative stress cannot be avoided. Moreover, ROS accumulation tends to increase over time, while defense mechanisms against ROS decrease. This condition may accelerate the aging process and lead to several pathological conditions such as inflammation, skin cancer, melanogenesis, and the alteration of sebum secretion, further accelerating skin aging. To delay this condition, supplementation of exogenous antioxidants may be relevant to support the natural defense mechanisms in protecting skin cells from damage and aging, as well as improving the quality and appearance of the skin.

The rationale for the antioxidant therapy is to balance the ROS-neutralizing activity with ROS generation. This goal can be achieved by enhancing the endogenous antioxidant system and the radical scavenging activity of exogenous antioxidants (Figure 2). Various polyphenols, including resveratrol, epicatechin, and rutin, have been identified for their beneficial effects on skin health as potent antioxidants through radical scavenging mechanisms [40]. The effective concentration required to scavenge 50% 2,2-diphenyl-1-picrylhydrazyl (DPPH) free radicals (EC_50_) range from 0.08 to 0.25 micromoles. This scavenging activity is significantly higher than that of Trolox as an antioxidant compound reference. According to a recent study, topical administration of a resveratrol solution mainly accumulates in the stratum corneum, with a gradual concentration on each layer from the upper to the lower layer [41]. It preserves its radical scavenging activity in a concentration-dependent manner. Another potent lipid-soluble antioxidant, vitamin E, exhibits potential free radical scavenging activity by disrupting the propagation reaction during the oxidation process [42]. The hydroxyl group in polyphenol compounds and vitamin E donates an electron or hydrogen atom, activating them for the scavenging of free radicals [43]. However, the stability issue of several antioxidant agents poses a significant challenge in their formulation to achieve the maximum benefits of antioxidant therapy.

Besides their direct free radical scavenging activity, some exogenous antioxidants may also support the activity of the endogenous antioxidant system. Topical administration of quercetin prevents glutathione (GSH) depletion during ultraviolet B (UVB) irradiation in hairless mice [44]. A similar recovery of intercellular GSH concentration has been found due to the induction of MRP1 expression during quercetin treatments, facilitating the rapid cellular export of quercetin–GSH conjugates [45]. Moreover, quercetin has also been found to boost the glutamate–cysteine ligase (GCL) expression, which is responsible for catalyzing the GSH synthesis. Another study reported that the upregulation of several endogenous antioxidants’ expression, including CAT, GPx1, SOD1, and SOD2, can be obtained during the treatment of foreskin fibroblast cells using a 50% ethanolic extract of *Centella asiatica* [46]. Additionally, *Centella asiatica* was found to downregulate the expression of matrix metalloproteinase 9 (MMP-9), which was upregulated during oxidative stress. MMP-9 is a zinc-containing gelatinase that degrades collagen in the skin, reducing elasticity and promoting wrinkles.

In a systematic review conducted by Sztretye et al., it was found that astaxanthin has effects on the skin, particularly in terms of addressing photoaging [47]. Photoaging results in the breakdown of extracellular matrix components such as collagen and elastin, leading to wrinkles, pigmentation changes, and a decline in skin texture. UV exposure stimulates the production of ROS in the skin. ROS oxidize the C8 guanine base, producing 8-hydroxy-2-deoxyguanosine (8-OHdG), a marker of DNA damage. In keratinocytes and fibroblasts, ROS activate cytokine receptors and growth factors, which trigger the mitogen-activated protein kinase (MAP kinase) pathway and activate the transcription factor activator protein-1 (AP-1). AP-1 enhances the expression of matrix metalloproteinases (MMPs), enzymes that degrade collagen in the skin. Ongoing carotenoid supplementation has been shown to provide protection against UV radiation, with astaxanthin, in particular, improving skin aging. It helps restore skin elasticity, reduce wrinkle formation, and maintain the integrity of the epidermal barrier.

## 5. Current Clinical Application of Topical Antioxidant Therapy

The use of topical antioxidant therapy in clinical settings relies on our current knowledge of how free radicals impact the physiological processes in the human skin. Extended exposure of free radicals to the skin can alter the composition of sebum and the integrity of the stratum corneum, while also exacerbating indications of skin aging by causing the development of pigmented spots and wrinkles [6].

The human skin naturally possesses defense systems to combat the generation of free radicals. Exogenous antioxidants may be necessary when the skin is consistently exposed to pollutants and radiation. Several compounds, both synthetic and organic, are known to have antioxidant effects and are, therefore, widely applied in topical cosmetics such as tocopherol (vitamin E), ascorbic acid (vitamin C), butylated hydroxy anisole (BHA), butylated hydroxy toluene (BHT), and propyl gallate. High levels of γ-tocopherol in the human skin play a role in protecting the epidermis from oxidative stress through the suppression of the synthesis of PGE2 and nitric oxide, reducing sunburn cell formation, peroxidation induced by UVB, and oedema. Over-the-counter antiaging creams typically include 0.5–1% vitamin E.

Vitamin C is a potent antioxidant in the skin that has demonstrated protective effects against photoaging, UV-induced immunosuppression, and photocarcinogenesis. It also exhibits antiaging properties by enhancing collagen production, stabilizing collagen fibers, and reducing collagen breakdown. It inhibits melanin production, leading to a decrease in pigmentation [48]. Vitamin C shields the skin from oxidative stress by delivering electrons in sequence to counteract free radicals [49]. Oxidized forms of Vitamin C are relatively unreactive [50]. Moreover, they can be transformed back into Vitamin C by the enzyme dehydroascorbic acid reductase in the presence of glutathione. UV radiation exposure decreases the presence of Vitamin C in the skin [49]. Although Vitamin C is equally effective against both UVB (290–320 nm) and ultraviolet A (UVA; 320–400 nm) [51], its UV protection can be optimized by combining it with sunscreens. Vitamin C does not absorb UV light but exerts a UV-protective effect by neutralizing free radicals, while sunscreens block 55% of the free radicals produced by UV exposure [49]. Vitamin C also becomes more effective when combined with Vitamin E, which enhances the effects of Vitamin C by four times [51]. This combination reduces chronic UV damage by decreasing cell apoptosis and thymine dimer production synergistically [52]. However, most cosmeceuticals with Vitamins E and C are ineffective when applied topically due to their instability upon exposure to air and light after opening [53].

BHA and BHT are synthetic antioxidants commonly utilized because of their low-cost production [54]. Both act by blocking the propagation of peroxy radicals [55]. However, research indicates that consuming excessive amounts of synthetic antioxidants can potentially be harmful to the health [56] due to their carcinogenic properties [57]. Although synthetic antioxidants are prevalent on the market, there has been a growing demand for natural antioxidants in recent years, and this trend is projected to persist [58].

## 6. Natural-Resource-Derived Antioxidants

There has been a shift in the demand for antioxidants in recent years. People now prefer naturally derived antioxidant agents over synthetic ones. Exogenous natural antioxidants can be regarded as bioactive compounds, typically sourced from plants. Natural antioxidants found in foods, like phenolic phytochemicals, appear to offer metabolic benefits and are linked to a reduced risk of various health issues [59,60,61]. This shift is supported by massive research on developing naturally derived antioxidant agents, particularly compounds derived from plants. Antioxidants that originate from nature have been identified as relatively safe ingredients, are affordable for most people, and meet the sustainability aspects [62]. Several secondary metabolites from plants have been identified as having robust antioxidant properties. Secondary metabolites can be defined as small organic molecules with a molecular weight of less than 3000 Da synthesized by plants as a self-defense mechanism to cope with environmental conditions [63,64]. Antioxidants play a critical role in plants, as they act as redox buffers interacting with various cellular components. This interaction influences the growth and development of plants by modulating processes ranging from mitosis and cell elongation to senescence and death [65]. Moreover, antioxidants impact gene expression associated with the stress response to optimize the plant adaptation through fluctuating conditions [66]. These valuable functions of plant-derived antioxidants can be further explored to obtain benefits for human health by extracting and isolating the antioxidant compounds contained in plants. Various factors affect the antioxidant activity of natural antioxidants. These include the plant species and variety, growing conditions, and extraction and processing techniques [67]. We summarize some of the potential natural resources for obtaining antioxidant activity (Table 1).

A research group in Thailand has studied 16 herbal extracts from various plant families to determine their antioxidant potential [68]. Extracts from *Phyllanthus emblica*, *Rosa damascene*, and *Stevia rebaudiana* were found to have the most significant antioxidant activity based on their radical scavenging activity and reducing power. Surprisingly, their activity was comparable to that of the ascorbic acid, which is widely recognized as a potent antioxidant. Another study reported the potential antioxidant activity of *Paeonia officinalis* leaf extract [69]. Based on an online screening of antioxidant capacity using high-performance liquid chromatography with a DPPH^•^ scavenging detector, it was discovered that gallic acid derivatives are the compounds responsible for the antioxidant activity. Additionally, the extract showed an inhibitory effect on α-amylase, with an enzyme inhibition activity (IC_50_) of 1.67 mg/mL. Furthermore, another research group from Iran investigated 20 species of plants belonging to the Lamiaceae family [70]. Their objective was to identify potential sources of antioxidants that can be further developed as raw materials for the cosmetic and pharmaceutical industries. The investigation involved in vitro antioxidant evaluation using DPPH and FRAP assays. Based on the results, two plants from the *Salvia* genus, namely *Salvia nemorosa* and *Salvia macrochlamys*, showed the highest antioxidant activity compared to the other species studied.

The protective effects of antioxidants in plants, including fruits and vegetables, are associated with three main categories: carotenoids, phenolic compounds, and vitamins [73]. Carotenoids are the second most prevalent natural pigments on Earth, in the form of lipophilic isoprenoid-based compounds found in all photosynthetic organisms, as well as some nonphotosynthetic bacteria and fungi. They range in color from colorless to yellow, orange, and red, giving fruits and vegetables their colors [74,75,76]. Found in all photosynthetic organisms and some autotrophic bacteria, carotenoids are not synthesized by animals and humans but are present in their blood and tissues as precursors to retinol (vitamin A). Typically, carotenoids are embedded in cell membranes as highly lipophilic molecules. Strict hydrocarbons like lycopene or β-carotene are located within the inner part of the lipid bilayer, whereas more polar carotenoids with attached oxygen atoms, such as lutein or zeaxanthin, align perpendicularly to the membrane surface with their hydrophilic portions facing the aqueous environment [77,78,79].

Carotenoids have attracted significant attention in recent years because of their potent antioxidant, reparative, antiproliferative, anti-inflammatory, and potential antiaging properties. They are useful in preventing diseases associated with oxidative stress and chronic inflammation [47]. Carotenoids, which are the precursors to vitamin A, are natural pigments characterized by highly conjugated π-bond systems, responsible for colors in fruits and vegetables. Carotenoids are classified into two main categories based on their structure: (i) carotenes, also known as carotenoid hydrocarbons, which consist solely of carbon and hydrogen, and (ii) xanthophylls, or oxygenated carotenoids, which contain various functional groups such as epoxy, methoxy, hydroxy, carbonyl, and carboxyl acid groups. Astaxanthin is one of the compounds that belong to the xanthophyll carotenoids and stands out as one of the most powerful carotenoids available [47].

Astaxanthin is a fat-soluble compound commonly found in microalgae, yeast, bacteria, and plants, but it is primarily present in algae and crustaceans, such as *Haematococcus pluvialis*, *Chlamydomonas nivalis*, fish, shrimp, fish eggs, and is also prevalent in other algae, plankton, and crustaceans, which is why it is often extracted from shrimp and crab shells, as well as other by-products [80,81]. Regular consumption of astaxanthin has many health benefits, such as strengthening and regulating the immune systems and reducing the risk of cardiovascular diseases [82], certain cancers, oxidative stress, inflammation, and several neurological disorders [83]. Additionally, astaxanthin provides protective effects for the skin [84], making it valuable for both the pharmaceutical and cosmeceutical industries.

The term vitamin E is used to describe eight lipophilic, naturally occurring compounds that include four tocopherols and four tocotrienols. All tocopherols and tocotrienols are powerful antioxidants that neutralize lipoperoxyl radicals through hydrogen donation from the phenolic group on the chromanol ring. Natural vitamin E forms with an unsubstituted 5-position, like γ-tocopherol, and can capture electrophiles, including reactive nitrogen species (RNS), which are elevated during inflammation. In contrast, vitamin E formed with a methyl group at the 5-position, such as α-tocopherol, does not have this capability [85,86,87]. Consequently, γ-tocopherol is more effective than α-tocopherol at detoxifying NO_2_ and peroxynitrite through the formation of 5-nitro-γ-tocopherol [88,89,90].

Medicinal plants have long been utilized to treat various illnesses due to their natural phytochemicals found in leaves, stems, fruits, flowers, roots, and bark. These phytochemicals include important antioxidant compounds such as flavonoids, phenolic acids, tannins, anthocyanins, and other phenolic compounds, which help combat oxidative effects [91,92,93,94]. For instance, rice contains phenolic and flavonoid compounds that contribute to its antioxidant properties. Seawan et al. found that pigmented rice varieties, such as red rice and black rice, exhibit a high antioxidant activity due to their rich content of bioactive compounds like phenolics, anthocyanins, and proanthocyanidins. Their research revealed that the average antioxidant activity measured using the DPPH method was 56.20 ± 3.92% for the black rice and 56.12 ± 1.28% for the red rice, both tested at a concentration of 5 μL of extract per 195 μL of 0.1 M DPPH solution [71].

In addition to carotenoids, vitamins, and phenolic compounds, there are other categories of substances recognized for their antioxidant properties, including essential oils. Essential oils are a great source of antioxidants that can be applied topically. These oils are extracted from different parts of plants using distillation or mechanical methods and are produced by glandular trichomes and other secretory structures in various plant organs [95]. Essential oils mainly comprise volatile compounds like monoterpenes and sesquiterpenes, produced through the mevalonate pathway [96]. Additionally, some oils contain phenolic compounds derived from the shikimate pathway. According to Chen et al., essential oils distilled from cinnamon and clove exhibit DPPH scavenging activity comparable with that of Trolox [72]. The antioxidant activity was likely derived from the high concentration of eugenol, a weakly acidic phenolic compound. On the other hand, the peppermint essential oil with the main component of menthol displayed a very weak antioxidant activity, with an EC_50_ value of 33.9 ± 5.64 mg/mL. This finding suggests that the main constituent of essential oils is the most significant factor in determining antioxidant activity.

Natural antioxidants are typically derived from natural sources such as plants. However, there are some limitations to using these sources, including a low yield of bioactive materials, variability in the concentration of bioactive materials due to geographical and environmental factors, and a complex and time-consuming extraction process. One solution to these challenges is using in vitro plant tissue cultures, which allow for a high yield of secondary metabolites with consistent and uniform quality, independent of geographic and environmental conditions [97]. In vitro plant tissue cultures produce secondary metabolites by growing and developing the plant cells, tissues, and organs using an artificial culture medium to supply nutrients under aseptic conditions [98]. This technique has successfully produced antioxidant bioactive metabolites from *Centella asiatica* [46]. Interestingly, the 50% ethanolic extract from *Centella asiatica* callus showed a higher antioxidant activity and a 2.5-fold more remarkable ability to neutralize free radicals than naturally grown plants. A similar technique has also been utilized to produce bioactive molecules from *Paeonia officinalis* var. *mascula* [99]. The bioactive molecules from the callus were extracted by sonication using water, followed by gradually increasing ethanol concentrations from 50% to 80%. Based on in vitro evaluation using human primary keratinocytes (NHEK cells), the pretreatment with the extract significantly protected the cells from the induction of oxidative stress. This protection mechanism was produced by modulating the mitochondrial function under oxidative stress and upregulating the expression of several genes implicated in epidermis function.

## 7. The Application of Nanotechnology in Topical Antioxidant Therapy

Nanocarriers have revolutionized antioxidant delivery by enhancing the solubility of drugs and providing better skin penetrability. Moreover, the nanocarrier system has optimized the delivery of drugs into the skin by increasing the skin permeation by the drug molecules [100]. Some nanocarriers also provide occlusive characteristics, which are beneficial to prevent water loss from the skin. Another potential advantage of nanocarriers is the ability to protect antioxidants from environmental degradation; thus, the stability of antioxidants can further be improved.

**Figure 4:**
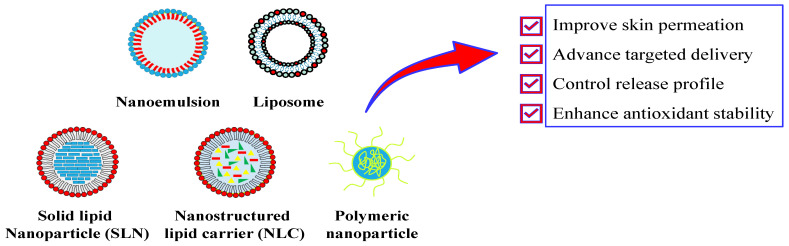
The illustration of the structure of nanocarrier systems for delivering antioxidants to the skin, including nanoemulsions, liposomes, SLNs, NLCs, and polymeric nanoparticles. The encapsulation of antioxidants into nanocarrier systems improves skin permeation, advancing targeted delivery, controlling the release of antioxidants, and enhancing antioxidant stability.

Several nanocarrier systems have been developed to deliver antioxidant molecules to the skin. Each nanocarrier system possesses its benefits and drawbacks relative to the other systems. Therefore, the selection of nanocarrier systems plays a crucial part in attaining optimal therapeutic outcomes. On the other hand, the characteristics of the compound should be considered to be another essential factor in determining the suitable nanocarrier system. For instance, a hydrophobic compound can be delivered using a lipid-based nanoparticle. On the other hand, hydrophilic molecules are better suited when encapsulated in a nanocarrier that offers an aqueous environment. Here, we outline five types of nanocarrier systems, with an emphasis on organic-material-based systems that have been extensively developed for delivering antioxidants to the skin through encapsulation mechanisms. These systems include nanoemulsions, liposomes, SLNs, NLCs, and polymeric nanoparticles (Figure 4). The application of a nanocarrier system to the delivery of antioxidants significantly enhances their protection against degradation during both storage and post-application. This advanced delivery system improves the permeation of antioxidants through the skin and increases their uptake at the cellular level. Moreover, the nanocarrier system facilitates the controlled release of antioxidants, optimizing topical antioxidant therapies’ effectiveness.

### 7.1. Nanoemulsions

Lipid-based formulations give rise to the most potent carrier system for delivering active molecules into the skin, given the similar lipophilic characteristics of the skin. One of the earliest lipid-based carrier systems to be developed is the emulsion system. This system consists of water and oil phases, in which one phase is dispersed in another phase and stabilized by emulsifying agents. The further development of the conventional emulsion has created a novel emulsion system with nanoscale globule sizes, namely nanoemulsions. These nanoemulsions are more attractive than conventional emulsions, especially in the cosmetic field, since the system has a clear and transparent appearance due to an extremely small globule size in the range of 20–200 nm [101]. Moreover, the nanoscale globule size significantly increases the contact surface area, a phenomenon which can facilitate the permeation process. The selection of the oil phase composition could improve the solubility of lipophilic drugs and could be utilized to control the drug release profile. There are at least three types of nanoemulsion systems, i.e., an oil-in-water nanoemulsion (O/W NE), in which the oil is dispersed in the water phase, a water-in-oil nanoemulsion (W/O NE), which is the opposite of the previous type, and a bi-continuous nanoemulsion [102]. Several antioxidants have been reported as being delivered using nanoemulsion systems (Table 2).

In the context of the antioxidant delivery system, nanoemulsions provide protection for the drugs against degradation. For example, by encapsulating the drugs inside the dispersed phase, nanoemulsions could reduce the enzymatic degradation and hydrolysis of the cargo by minimizing the contact of the drugs with the environment [103]. Furthermore, the entrapment of quercetin inside an O/W NE decreases the degradation rate from 42% in the water/ethanol system to 9% [104]. Quercetin is a phenolic compound found in abundant amounts in several plants. This potent antioxidant has a poor water solubility, making the formulation and delivery process become challenging. These problems can be tackled by formulation through a nanoemulsion system. Additionally, the DPPH scavenging activity results indicate that quercetin nanoemulsions demonstrate the highest free radical scavenging activity, with an EC_50_ value of 28.88 ± 1.00 µM. In comparison, curcumin nanoemulsions show an EC_50_ value of 45.53 ± 2.00 µM, while ascorbic acid nanoemulsions have an EC_50_ value of 51.51 ± 2.00 µM. Zorzi et al. reported the development of nanoemulsions containing quercetin as an isolated compound and from *Achyrocline satureioides* extract [105]. The nanoemulsion was built by spontaneous emulsification with egg lecithin, octyldodecanol, and water. From the skin permeation study using the back part of the porcine skin ear, the nanoemulsion showed a good ability to retain the quercetin in the skin. Interestingly, the nanoemulsion containing the extract displayed more quercetin retention in the skin than the nanoemulsion containing the quercetin isolate by 2.5 folds.

Ruktanonchai et al. reported the encapsulation of alpha-lipoic acid into several lipid-based nanocarriers, including nanoemulsions, NLCs, and SLNs [106]. The nanoemulsion was created by combining 9% of Miglyol 812^®^ as a lipid phase and 2.5% of Pluronic^®^ F68 as a surfactant. The resulting globules had a hydrodynamic diameter of 113 ± 12 nm and a zeta potential of −27.8 ± 2.6 mV. The in vitro release study using Franz diffusion cells revealed that the nanoemulsion showed the highest release of alpha-lipoic acid, with 62% after 72 h compared to NLCs and SLNs. The higher release rate from the nanoemulsion system was due to the nanoemulsion component consisting of liquid lipids compared to NLCs and SLNs, both of which consist of solid lipids; therefore, the viscosity was relatively lower, and the diffusion process of the drug could be higher. Clares et al. also reported a similar result using a lipophilic antioxidant of retinyl palmitate [107]. In this study, retinyl palmitate was delivered using a nanoemulsion, a liposome, and a SLN. Interestingly, the nanoemulsion showed the smallest particle size, with a diameter of 14.42 ± 1.10 nm, compared to 176.53 ± 1.67 nm and 271.5 ± 2.4 nm for the liposome and the SLN, respectively. Based on the permeation study, the retinyl palmitate delivered by the nanoemulsion exhibited the highest skin penetration level compared to the liposome and the SLN. The presence of a surfactant and a co-surfactant in nanoemulsion formulations may be used to exemplify the significant enhancement in permeation ability [108]. Therefore, optimization should be performed to select the best composition of surfactants and co-surfactants to have a delivery process suitable for therapeutic purposes.

In recent years, nanoemulsions have been developed as potential carriers for delivering essential oils. These oils have volatile characteristics and contain various chemical constituents that are beneficial to the health, one of which has the potential to be an antioxidant [96]. Commonly, essential oils have a unique aroma that makes them very attractive for application in cosmetic products. However, the direct contact between essential oils and the skin could induce allergic contact dermatitis and increase the risk of skin irritation [109]. These negative impacts of using essential oils may be reduced by solubilizing or dispersing the essential oils into the dispersed phase of a nanoemulsion so that direct skin contact can be minimized. Several potential essential oils have been reported as being delivered using nanoemulsions, including lemon oil, ginger oil, almond oil, and neem oil [110,111,112]. From the reported data, nanoemulsions could preserve the antioxidant activity of essential oils, as well as significantly lessening the skin irritation effect. Attention to incorporating essential oils into the nanoemulsion system is crucial, especially when producing nanoemulsions that require a heating process.

**Table 2:** List of antioxidants delivered by nanoemulsion.

Cargo	Lipid	Surfactant	Result	Refs.
Quercetin	Lemon oil and corn oil	Saponin and Tween 80	Particle size: 52.0 ± 10.0 nm ζ-potential: −41 ± 8 mV	[104]
Quercetin	Egg lecithin	Octyldodecanol	Particle size: 197.0 ± 10.0 nm ζ-potential: −27.4 ± 6.0 mV	[105]
*Achyrocline satureioides* extract	Egg lecithin	Octyldodecanol	Particle size: 295.6 ± 9.0 nm ζ-potential: −43.6 ± 2.1 mV	[105]
Alpha-lipoic acid	Miglyol 812^®^	Pluronic^®^ F68	Particle size: 113.0 ± 12.0 nm ζ-potential: −27.8 ± 2.6 mV	[106]
Retinyl palmitate	Labrafac^®^ lipophile	Labrasol^®^ and Plurol^®^ oleique	Particle size: 14.4 ± 1.1 nm ζ-potential: n.a.	[107]
Lemon oil	Lemon oil	Tween 80 & Span 80	Particle size: 64.6 ± 1.6 nm ζ-potential: n.a.	[110]
Ginger oil	Ginger oil	Tween 80 and ethanol	Particle size: n.a. ζ-potential: n.a.	[111]
Almond oil	Almond oil	Tween 80	Particle size: 114.1 ± 3.8 nm ζ-potential: −6.8 ± 0.2 mV	[112]
Neem oil	Neem oil	Tween 80	Particle size: 73.4 ± 7.6 nm ζ-potential: −16.2 ± 0.5 mV	[112]

n.a. = not available.

### 7.2. Liposomes

**Figure 5:**
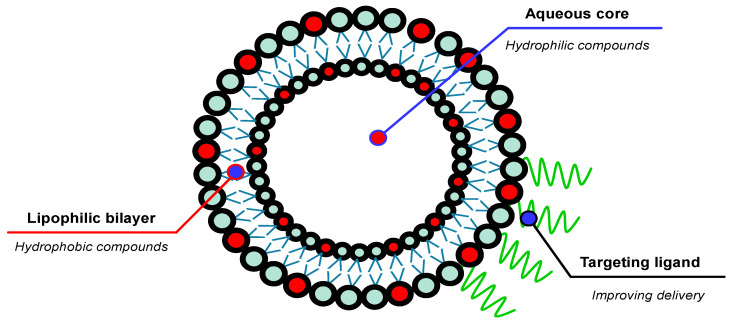
Schematic representation of the liposomal nanocarrier, consisting of the lipophilic phospholipid bilayer to facilitate the encapsulation of hydrophobic compounds, with the hydrophilic core for entrapping the hydrophilic molecules. The targeting ligands could be embedded into the membrane bilayer to modify the surface properties of the liposome.

Liposomes are spontaneously formed spherical vesicles composed of one or more phospholipid double layers that enclose the hydrophilic aqueous core with a size ranging from 30 nm to several micrometers [113]. This system was first introduced by Alec D. Bangham and his team in the 1960s [114]; nowadays, it has evolved into the most valuable drug-carrier system for several biomedical applications, including cancer therapy [115], gene therapy [116], immunotherapy [117], and antioxidant carrier [118]. Since it has biphasic properties, liposomal nanocarriers enable the encapsulation of both hydrophilic and hydrophobic compounds. Moreover, the flexibility of the liposomal bilayer membrane allows it to be modified with several targeting ligands to improve the delivery efficiency [119] (Figure 5). Numerous techniques have been developed for manufacturing liposomes, ranging from traditional methods, such as the thin-film hydration method, the detergent removal method, the solvent injection method, and the reverse-phase evaporation method, to more novel approaches, including lyophilization, supercritical fluid-assisted methods, microfluidic techniques, and membrane contractor methods [120]. In the skin drug delivery system, liposomes exhibit an enhancement in drug penetration capacity through the skin, resulting in significantly higher drug concentrations in the epidermis and dermis with relatively lower drug systemic availability compared to the conventional topical dosage forms [121]. To produce a more rigid and stable liposomal structure, cholesterol is usually added to the liposome formulation, since cholesterol could increase the packing of phospholipid molecules [122,123]. The first liposomal cosmetic product for antiaging was introduced by Christian Dior in 1986 under the brand name Capture. Table 3 summarizes several topical antioxidant delivery systems that utilize the liposomal technology.

Ascorbic acid or vitamin C is one of the water-soluble vitamins that have been widely utilized as a potent antioxidant. Vitamin C has been reported to have several beneficial effects on the skin, including preventing skin photodamage [130], supporting collagen synthesis [131], lowering the pro-inflammatory cytokines such as TNFα, IL-1, IL-6, and IL-8 [132], and slowing down skin hyperpigmentation by inhibiting the tyrosinase [52]. Despite the several functions of vitamin C in skin health, this molecule is prone to photooxidation. Moreover, vitamin C struggles to penetrate the stratum corneum due to its hydrophilic characteristics. The encapsulation of the vitamin C into a liposomal system has been reported to engender a significant improvement in human skin penetrability [124]. The improvement is caused by the impact of the hydrophobicity of the liposomal system, which can further improve the interaction with corneocytes. Furthermore, the liposomes containing vitamin C show a better antioxidant activity and anti-inflammatory effect in the human skin exposed to UVA/UVB radiation. A significant enhancement in skin penetration and epidermal accumulation has been observed when an antioxidant complex, comprising ectoin, the microalgae extract *Haematococcus pluvialis*—rich in astaxanthin—and tetrahexyldecyl ascorbate, is encapsulated within a liposomal system containing unsaturated phosphatidylcholine [125]. This liposomal system treatment has been shown to result in the normalization of interleukin-6 (IL-6), interleukin-8 (IL-8), and matrix metallopeptidase-9 (MMP-9) back to their baseline levels following exposure to irradiation.

Astaxanthin, a natural lipophilic ketocarotenoid that contributes to the pink color of salmon, is well known for its exceptional antioxidant properties [133]. Despite its potential benefits, the efficacy of astaxanthin is hindered by its poor water solubility, unstable nature, and low bioavailability. To enhance the stability of astaxanthin, Luo et al. developed a liposomal system composed of soy lecithin and cholesterol in a 10:6 ratio [126]. Using the ethanol injection method, they successfully encapsulated astaxanthin within the liposome structure, achieving a relatively high encapsulation efficiency of approximately 88%. However, this liposomal encapsulation reduced astaxanthin’s antioxidant activity compared to its ethanolic solution. Nevertheless, the liposomal system demonstrated a significant improvement in stability, with about 95% of astaxanthin remaining stable within the liposomes, in contrast to the 72% retention observed in the ethanolic solution. Another research group explored the use of chitosan as a coating material for astaxanthin liposomes to further enhance antioxidant activity [127]. They created a core-shell nanostructure using astaxanthin liposomes as the core. These liposomes were prepared through the thin-film hydration method, which involved the combination of soy lecithin and cholesterol in a 10:2 ratio. Following this, the astaxanthin liposomes were coated with chitosan, forming the shell structure by employing a high-pressure homogenization method. Chitosan can attach to the surface of astaxanthin liposomes through the formation of hydrogen bonds, as demonstrated by FT-IR spectroscopy data. The chitosan-coated astaxanthin liposomes displayed an enhanced antioxidant activity compared to their uncoated counterparts. However, it is important to note that the chitosan coating reduced the release rate of astaxanthin, as the chitosan increased the mechanical rigidity of the liposomes’ surface.

The encapsulation of the phenolic antioxidant, caffeic acid, into a liposomal system has been achieved using a reverse-phase evaporation method with a relatively high encapsulation efficiency of 70% [128]. The resulting particles displayed a good stability during the storage at 4 °C for two months, which was indicated by a negligible change in particle size and zeta potential. The caffeic acid encapsulated in the liposomes exhibited an almost eight times higher epidermal penetration compared to the free caffeic acid, with 41.8% of caffeic acid transferred through the pig ear epidermis after 7 h and an average permeability of 28.19 × 10^−3^ cm min^−1^. Phosphatidylcholine, as the main lipid, was identified as the penetration enhancer through the interaction with and the hydration effects on the stratum corneum. Another research group reported encapsulating a lipophilic endogenous antioxidant, coenzyme Q_10_ (CoQ_10_), into a liposomal nanocarrier consisting of phospholipid-Lipoid S100 and cholesterol [129]. The antioxidant activity was evaluated in the normal human fibroblast cells by induction of oxidative stress using H_2_O_2_. The pre-treated cells using the CoQ_10_ liposome exhibited more resistance to oxidative stress than free CoQ_10_ and CoQ_10_-loaded SLNs, as indicated by a significantly higher cell viability after the induction of oxidative stress. The result was also in line with the cellular ROS level, which decreased by 50% compared to the negative control. Moreover, the optimum CoQ_10_ concentration to protect against oxidative stress adequately was 50 μM.

Clares et al. reported a comparison study of a hydrophobic vitamin A derivative, i.e., retinyl palmitate, delivered using several lipid-based nanoplatforms, including nanoemulsions, liposomes, and SLNs [107]. Liposomal delivery of retinyl palmitate demonstrated the best skin retention relative to nanoemulsions and SLNs. Prolonged skin retention could be due to biocompatible phospholipids, which could interact adequately with the skin. In the context of drug permeation and flux, liposomes exhibited an intermediate level, corresponding to nanoemulsions and SLNs, with 4.36 ± 0.21 μg of retinyl palmitate penetrating into the skin during 38 h with a 0.15 ± 0.09 μg/h flux. Moreover, the liposomal nanocarrier displayed the hydration effect characterized by extensive interlamellar gaps between the stratum corneum and the uppermost layer of the epidermis. The surface modification of the liposomes using polyethylene glycol (PEG) could further improve the transdermal flux and skin hydration [134]. The PEG molecules bind to the water molecules, auguring skin hydration and enhancing permeability across the stratum corneum. However, technological problems, particularly in the scale-up process, are still the main challenges in developing liposomal nanocarrier as a drug delivery platform for clinical use.

### 7.3. Solid Lipid Nanoparticles (SLNs)

In the early 1990s, Müller and co-workers successfully created a novel lipid-based nanocarrier, namely a solid lipid nanoparticle (SLN), as an alternative to overcome the technological problems of mass production and the safety issues of the other existing colloidal dispersion systems, including liposomes, nanoemulsions, and polymeric nanoparticles. The idea behind developing this nanocarrier system is that many solid lipids have been developed as well-tolerated drug delivery systems, and large-scale production may be efficiently conducted using high-pressure homogenization [135]. SLNs are composed of inert lipid materials, which are generally recognized as safe (GRAS) by the regulation, dispersed in an aqueous surfactant solution with particle sizes ranging from 50 nm to 1000 nm in which the dispersed lipids are in solid form at room and body temperature [136]. By using solid lipid materials, SLNs could control the drug release by lowering the drug mobility in the matrix and improving the stability of the cargo [137,138]. Moreover, SLNs provide an occlusive effect, which is essential to increase skin hydration and facilitate skin penetration [139]. The overall properties of SLNs provide these lipid-based nanocarriers with the potential to deliver antioxidants to the skin (Table 4).

A lipophilic antioxidant, i.e., resveratrol, has been encapsulated into an SLN containing Compritol^®^ 888 ATO as the solid lipid and a combination of Poloxamer^®^ 188 and Tween^®^ 80 as the surfactant system using the high-pressure homogenization method [140]. The optimum particle was constructed with a solid lipid, surfactant, and drug composition ratio of 30:22.5:1, with an encapsulation efficiency of 73% and a particle size of around 287 nm. From the ex vivo evaluation using Franz diffusion cells with rat abdominal skin, it was found that the SLN could improve the delivery of resveratrol into the skin with preferential accumulation in the epidermis. The encapsulation of the other poorly water-soluble antioxidant, i.e., alpha-lipoic acid, into an SLN system comprising 1% Apifil produced a prolonged release, with a release rate constant of 3.206 μg cm^−2^ h^−1/2^ [106]. The release rate observed was slower than that of nanoemulsions and nanostructured lipid carriers (NLCs), primarily due to the presence of a solid lipid matrix that increases the viscosity, thereby reducing the diffusion rate. This slower release characteristic may be advantageous for depot systems, as it ensures minimal fluctuations in drug concentrations at the targeted site, thereby enhancing patient convenience.

SLNs possess the capability not only to regulate the release of drugs, but also to protect them against photodegradation. Mitri et al. reported on the encapsulation of lutein, a lipid-soluble antioxidant, in an SLN containing 9% carnauba wax as a solid lipid matrix core using the high-pressure homogenization method [141]. The lutein-loaded SLN displayed the most significant enhancement in photostability relative to NLCs and nanoemulsions, with only 0.06% lutein degradation after irradiation with 10 MED (minimum erythema dose). The photostabilizing effect was possibly due to the crystalline lipids that can reflect the ultraviolet radiation, and some lipids like carnauba wax behave as a molecular sunscreen. A similar photoprotective effect has been obtained with tretinoin encapsulated in an SLN composed of a glyceryl monostearate matrix core [142], as well as with retinyl palmitate in Compritol^®^ 888 ATO [107]. The protection effect against factors that cause oxidation is critical, particularly in delivering oxidation-sensitive molecules like antioxidants.

To further improve the skin permeation capacity, surface modification with several surface modifiers has been conducted. One of the surface modifiers utilized is dicetyl phosphate (DCP) [143]. This molecule modifies the surface of SLNs to become negatively charged. Compared with neutral charge SLNs, DCP-modified SLNs significantly improve the delivery of retinyl palmitate into the skin by a factor of 4.8. Furthermore, most drugs are retained in the epidermal layer, making SLNs a potential topical carrier with minimal systemic effects. This result is also in line with a report from Montenegro et al., who encapsulated a synthetic ubiquinone derivative into an SLN with a predominant accumulation in the upper layer of the skin [144]. Moreover, the encapsulation of pyroglutamic acid ester form of idebenone (IDEPCA) into an SLN system using the phase inversion temperature (PIT) method has also been reported to have superior skin hydration effects compared to the SLN-encapsulated parent drug [145]. This enhancement in skin hydration is attributed to the synergistic interplay between the ester derivatives and their encapsulation within the SLN system.

Since the core matrix of solid lipid nanoparticles (SLNs) comprises lipids, this system is particularly effective for encapsulating hydrophobic compounds. Nonetheless, there have been several attempts to incorporate hydrophilic compounds into SLN systems. One approach involved the use of a double emulsion/melt dispersion technique [146]. The particle size of the SLN system was mainly controlled by the lipid amount and the type of surfactant system used. Another method utilized a thermoresponsive gel core made from polymers to encapsulate hydrophilic molecules, which were then coated with lipid components to create the SLN system [147]. Although the process of encapsulating hydrophilic substances into the SLN system is possible, the approach used has the potential to impact on the characteristics of the resulting SLN, one of which is the drug release process from the SLN. Utilizing both approaches mentioned earlier hinders the drug release process due to the requirement for a partitioning process between the aqueous matrix or hydrogel core and the lipid core of the solid lipid nanoparticles (SLNs). Thus, it is recommended to use other types of nanocarriers that can accommodate the encapsulation of hydrophilic compounds, such as liposomes and polymeric nanoparticles.

**Figure 6:**
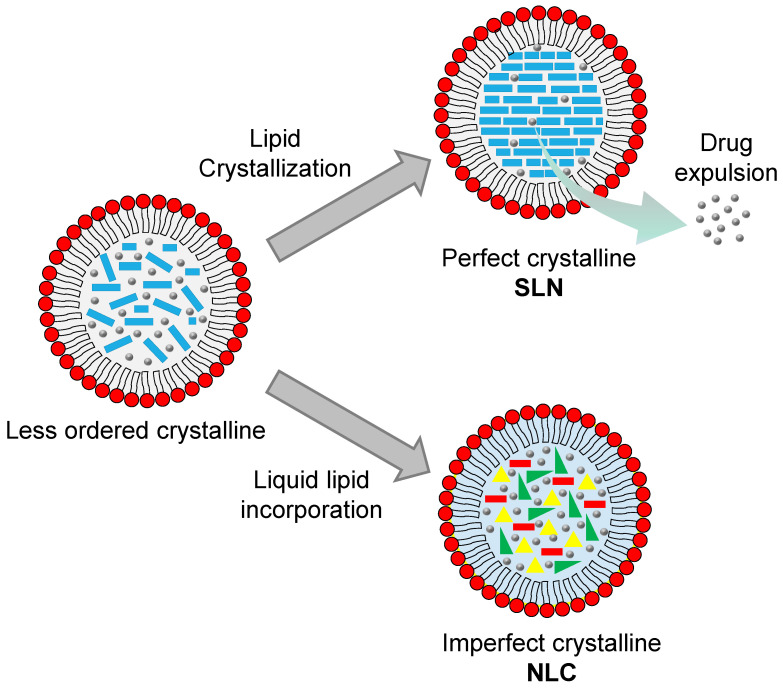
Mechanism of drug expulsion from SLNs during lipid crystallization. The formation of a perfect crystalline arrangement in SLNs discharges the drug from the core, thus declining the encapsulated drug. The incorporation of liquid lipids into NLCs averts the formation of a highly ordered crystalline structure in the core, maintaining the encapsulated drug.

Despite the several benefits offered by SLNs as the carriers for skin delivery systems, SLNs display several shortcomings. Due to the use of solid lipids as the core matrix, SLNs tend to form an ordered internal structure as a result of the crystallization process of the lipids during the preparation and storage. This crystallization process lowers the drug loading capacity and causes the drug expulsion from the core matrix during storage (Figure 6). Furthermore, the high water content of SLNs, ranging from 70–99.9%, could be a problem in incorporating SLNs into the final topical products like creams or ointments [148,149]. Incorporating liquid lipids into the SLN structure overcomes these drawbacks, with such structures then known as nanostructured lipid carriers (NLCs).

### 7.4. Nanostructured Lipid Carriers (NLCs)

Nanostructured lipid carriers (NLCs) are the second generation of lipid nanoparticles. These nanocarriers are developed to further improve the performance of SLNs. To reduce the negative effect of lipid crystallization in SLNs, liquid lipids are introduced into the NLC formulation. The presence of liquid lipids distorts the formation of a perfect crystalline core, resulting in a distant structure. Furthermore, it alters the internal structure of NLCs, from the ordered crystalline core of SLNs to a less ordered crystalline arrangement (Figure 6), preventing drug leakage during storage [150]. The addition of liquid lipids also enhances the solubility of some drugs, thus further increasing the drug loading efficiency [151]. Therefore, NLCs are widely applicable to the delivery of poorly water-soluble drugs through several primary administration routes, including parenteral, oral, ocular, nasal, and topical. Basically, NLCs comprise a combination of solid and liquid lipids dispersed in an aqueous phase that is stabilized with a suitable surfactant system. Since the liquid lipids lower the viscosity and the interfacial tension of the mixtures, replacing some solid lipids with liquid lipids may reduce the particle size [140,152]. The lipid components provide an occlusive effect on the skin, promoting drug penetration and better skin hydration.

A significant improvement in skin delivery of α-tocopherol was achieved by incorporating it into an NLC [153]. The encapsulation process was carried out using high-pressure homogenization with tripalmitin, oleic acid, and Tween 80 as the solid lipid, liquid lipid, and surfactant, respectively. The resulting particles showed a homogenous particle distribution with a hydrodynamic size of around 67 nm. The α-tocopherol-loaded NLC exhibited a controlled drug release profile, with 30% and 75% drug release after 2 h and 24 h, respectively. The α-tocopherol predominantly accumulated in the epidermis layer, with a concentration of 762.3 ± 184.6 ng/mL compared to 182.3 ± 52.7 ng/mL when delivered by nanoemulsion. The skin delivery of CoQ_10_ using an NLC has also been reported to promote a significant advancement in the photoprotective effect by reducing lipid peroxidation and cellular ROS levels as well as improving SOD and glutathione peroxidase (GSH-px) activity [154]. A significant augmentation of CoQ_10_ activity resulted from an improved skin penetration ability. The NLC system could deplete the barrier function of the stratum corneum, characterized by swelling and thickening of the stratum corneum [155]. Furthermore, the presence of a surfactant on the surface of the NLC structure further enhances the disruption effect on the stratum corneum and facilitates the denaturation of keratin; thus, NLCs could passively permeate the skin [156].

Several other natural antioxidant compounds have been reported to be delivered into the skin using an NLC system, including quercetin, lutein, and curcumin. Natural antioxidants display a strong potential to prevent oxidative stress by controlling the formation of ROS and scavenging free radicals [157]. Despite the benefits of natural antioxidants, most of the substances in this class exhibit low percutaneous permeation and poor aqueous solubility, thus making the formulation and topical delivery process challenging. Pivetta et al. reported encapsulating the flavonoid antioxidant of quercetin into an NLC system using the emulsion and sonication method [158]. The resulting particles displayed a high encapsulation efficiency of around 98% due to the high affinity of the compound with the lipid matrix containing illipe butter and calendula oil. The quercetin-loaded NLC also showed a homogenous particle size distribution, with an average size of 130 nm and good stability, indicated by the negligible change in quercetin concentration inside the NLC after 90 days of storage. The low recrystallization index of 13.03% can be used to explain the good stability profile of the NLC.

Another potential xanthophyll carotenoid antioxidant, i.e., lutein, has been encapsulated into an NLC containing palmitic acid, Lipoid S100, and FloraGLO^®^ using a solvent injection method [159]. This technique has been claimed to reduce the use of surfactants and avoid the heating process of the drugs; therefore, it is suitable for thermolabile molecules. The resulting particles displayed a higher skin permeation ability relative to the lutein suspended in an oil vehicle. A similar trend in skin permeation ability has also been displayed by a curcumin-loaded NLC, with 22.06 ± 1.59% of curcumin successfully permeating the skin within 24 h [160]. Curcumin is a potent polyphenol antioxidant derived from *Curcuma longa*, with highly lipophilic characteristics and a high sensitivity to oxygen and light. To facilitate its application to the skin, the curcumin-loaded NLC was then incorporated into the Carbopol^®^ 980 NF gel matrix. However, the percutaneous permeation was significantly reduced by a factor of 4.3 compared to the nonincorporated particles due to the lower mobility of the NLC inside a highly viscous gel matrix. The reduction in the permeation ability may be exploited to obtain a controlled release for a prolonged effect. Furthermore, the viscosity of the matrix vehicle should be considered as the predominant aspect to have a suitable release rate.

In addition to delivering the natural antioxidant as an isolated compound, NLCs can also potentially deliver essential oils. A study found that clove essential oil (CO) can be encapsulated into an NLC system containing beeswax, virgin coconut oil (VCO), Tween 80, and propylene glycol with a high encapsulation efficiency and stability [161]. The process of encapsulation using the emulsification sonication method also preserved the free radical scavenging activity of the CO. The study found that the CO-loaded NLC could protect fibroblast cells from oxidative stress induced by H_2_O_2_, while free CO was ineffective at rescuing the cells from oxidative damage. This finding verifies that the NLC system is a promising drug delivery strategy for essential oils with unique properties like high volatility, low aqueous solubility, and potential skin irritation effects.

### 7.5. Polymeric Nanoparticles

**Figure 7:**
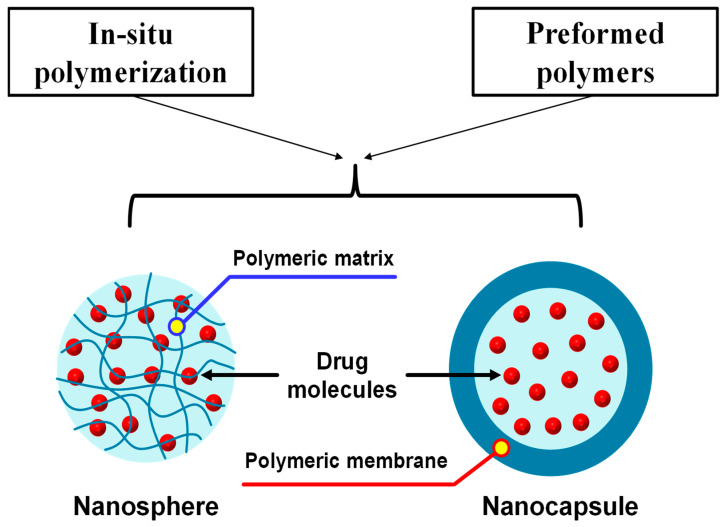
The classification of polymeric nanoparticle systems, consisting of nanospheres and nanocapsules. Nanospheres consist of a continuous polymeric structure, while nanocapsules comprise a polymeric shell in which the drug can be incorporated inside. These polymeric nanoparticles can be synthesized through the in situ polymerization of monomers and the direct utilization of preformed polymers.

Several polymers can be utilized to develop a nanocarrier system for delivering drugs to a specific body site, namely polymeric nanoparticles. The size of the resulting particles can be engineered in the range of 1 to 1000 nm. Polymeric nanoparticles have drawn much attention as a potential nanocarrier system for delivering a wide range of biorelevant molecules, including small chemical molecules, macromolecules, and genetic materials [162] for several targeting purposes. Based on the morphology of the particles, the polymeric nanoparticles can further be divided into nanospheres and nanocapsules [163] (Figure 7). The drug molecules in a nanosphere are retained inside or adsorbed onto the surface of a continuous polymeric structure [164]. In contrast, the drugs are encapsulated inside the polymeric shell in a nanocapsule system [165,166]. Polymeric nanoparticles can be synthesized using two different methods, i.e., the polymerization of monomers and the direct utilization of the preformed polymers [167]. To tackle the safety issues of the nanosized materials, several biocompatible and biodegradable polymers have been synthesized and utilized as the main building block of the polymeric nanoparticles, including polylactic acid, poly lactic-*co*-glycolic acid, and PEG [168].

In the context of percutaneous delivery, polymeric nanoparticles offer several benefits, including the alteration of the physicochemical properties of the drugs, the controlled drug release profile, facilitated skin permeation, and increased skin adhesivity for prolonged contact [169]. Kim et al. reported on the encapsulation of retinol into a natural polymer of low molecular weight, i.e., water-soluble chitosan, through an ion complex mechanism between the amine group of chitosan and the hydroxyl group of retinol [170]. The encapsulation process resulted in a significant increase in retinol solubility by a factor of about 1600. In addition, the particle size significantly depends on the amount of retinol added; higher retinol levels result in larger particle sizes. A remarkable improvement in photochemical stability has also been reported for the encapsulation of retinol into chitosan nanoparticles [171]. An improvement in the drug release profile of quercetin has also been reported for the encapsulation in a combination of Eudragit^®^ E and polyvinyl alcohol (PVA) as the polymeric carriers [172]. The nanoparticles were prepared using a nanoprecipitation method. Using a quercetin, Eudragit^®^ E, and PVA ratio of 1:10:10 yields homogeneous particles approximately 82 nm in size, with an encapsulation efficiency of 99%. The combination of particle size reduction, amorphous transformation, and intermolecular hydrogen bonds resulted in a notable improvement in the antioxidant activity by a factor of 883 compared to quercetin dispersed in water. The results demonstrate the potential of polymeric nanoparticles to enhance the activity of antioxidants, a phenomenon which can be attributed to the improved solubility of water-insoluble antioxidants.

The encapsulation of antioxidant molecules into a polymeric nanoparticle system could further improve the protective effect against degradation, as demonstrated by the encapsulation of curcumin into cellulose-derived polymers. The stability of the curcumin against light and the oxidizing environment was significantly improved [173]. The nanospheres containing the curcumin were constructed using the self-assembly of polymer method, using ethyl cellulose and the combination of ethyl cellulose and methylcellulose. Furthermore, a better radical scavenging activity was obtained when the curcumin was delivered using polymeric nanoparticles compared to the free curcumin due to the better skin penetration ability. Another research group from Japan has reported on the application of an amphiphilic graft copolymer known as Soluplus^®^ (polyvinyl-caprolactam–polyvinyl-acetate–polyethylene-glycol graft copolymer) as a potential candidate for delivering various antioxidants [174]. They discovered that encapsulating nobiletin, a polymethoxy flavonoid, in polymeric nanoparticles containing Soluplus^®^ and l-ascorbyl 2,6-dipalmitate, using the hydration method, significantly improved skin penetration and the accumulation of nobiletin. The resulting polymeric nanoparticles also demonstrated a promising free radical scavenging activity, with an EC_50_ value of 46.7 µg/mL as evaluated by the DPPH scavenging test. In a recent report, the same group has detailed the encapsulation of astaxanthin within a Soluplus^®^ polymer, revealing that astaxanthin was retained in the stratum corneum [175]. Interestingly, when PEG 2000 was added to the formulation, astaxanthin was found in deeper layers within the epidermis. These findings indicate that polymeric nanoparticles serve to improve the skin penetration ability of antioxidants, as well as providing protection from environmental factors.

Polymeric nanoparticles have also been reported to have the potential to deliver plant-derived antioxidants in the form of extract. The biodegradable polymer of polycaprolactone (PCL) has been used to encapsulate the roasted leaves extract of *Ilex paraguariensis* using the double emulsion solvent evaporation technique [176]. The extract of *Ilex paraguariensis* was reported to have a high content of polyphenolic compounds, which have the potential for antioxidant activity. The nanoparticles were stabilized using a block co-polymer of Pluronic. Interestingly, the chlorogenic acid, a marker molecule of the *Ilex paraguariensis* extract, displayed a higher retention level in the epidermis than in the dermis relative to the free extract solution. It may be due to the particle size of the nanoparticle being too large to undergo passive diffusion through the skin structure compared to the molecularly dispersed free extract in the water. This result suggests the potential application of polymeric nanoparticles as carriers for cosmetic applications.

## 8. The Clinical Implementation of Nanotechnology for Topical Antioxidant Therapy

Various nanocarriers, including liposomes, NLCs, and nanoemulsions, are frequently used to transport antioxidants. The majority of the published clinical studies aim to demonstrate the effectiveness and safety of this nanoformulation in patients with melasma. Melasma is a persistent skin disorder characterized by hyperpigmentation caused by oxidative stress. Various antioxidants have been utilized to treat this hyperpigmentation disorder [177].

In a double-blind randomized clinical trial involving 20 women, liposomal hydroquinone exhibited a significant therapeutic effect on melasma based on the melasma area and severity index (MASI) [178]. However, it showed no superiority over conventional hydroquinone formulations. Another study on 23 melasma patients demonstrated that both liposomal tranexamic acid (TA) and conventional hydroquinone lead to significant reductions in mean MASI scores, with a slightly greater but statistically nonsignificant decrease observed with 5% liposomal TA [179]. Notably, irritation occurred in three patients using hydroquinone, while no serious adverse events were reported with TA. Additionally, a study on 21 female patients with melasma evaluated the effectiveness of a cream containing liposome-encapsulated 0.1% 4-n-butylresorcinol (4nBR) and resveratrol (RSV) [180]. The results demonstrate the cream’s efficacy and safety, with noticeable effects appearing as early as 2 weeks, confirming the synergistic effect of 4nBR and RSV in inhibiting melanogenesis. These findings help clarify how nanotechnology might be used to generate different formulations for treating melasma.

Various studies have explored the potential benefits of topical formulations in addressing diverse skin conditions associated with free radical involvement. For instance, a study investigating patients undergoing radiotherapy for cancer and healthy individuals revealed that a vitamin-E-containing nanoparticle cream exhibited a protective effect against radiodermatitis onset time in patients not receiving a boosted radiation dose, along with mild inframammary erythema occurrence [181]. No adverse effects were reported. A study focused on developing NLCs with a high skin deposition and strong antioxidant properties for utilizing the melatonin hormone and antioxidant oils to treat androgenic alopecia (AGA) found that melatonin NLCs had a nanometer size, a negatively charged surface, a high entrapment efficiency, potent antioxidant capabilities, and provided sustained release for 6 h [182]. In addition, NLCs exhibited an excellent storage stability and significantly enhanced the skin deposition of melatonin by a factor of 4.5 in the stratum corneum, by a factor of 7 in the epidermis, and by a factor of 6.8 in the dermis compared to a melatonin solution. The melatonin nanostructured lipid carriers (NLCs) showed superior outcomes in AGA patients compared to the melatonin solution, with higher hair density and thickness and reduced hair loss.

An in vitro study followed by an in vivo human study showed that hydrogel formulations including vitamin-E-loaded nanostructured lipid carriers (HG-NLCVE) had superior occlusive qualities, while the vitamin-E-loaded nanoemulsion (HG-NEVE) resulted in a quicker skin hydration effect [183]. Moreover, the latter was chosen as the most appealing for skin applications, while the HG-NLCVE was deemed to be more appropriate for achieving a prolonged impact. This work showed the safety and moisturizing properties of hydrogels with vitamin-E-loaded lipid-based nanosystems in laboratory and living organisms.

Natural resources have been used to create nanoformulations for clinical trials. A study was conducted on 40 male participants aged 18–28 years, separated into three groups: vehicle formulation, *Melaleuca alternifolia* pure essential oil, and *Melaleuca alternifolia* [184]. Nanoemulsions with *Melaleuca alternifolia* essential oil can improve photoaged skin by penetrating to the deeper layers of the skin. The nanoemulsion had a reduced antioxidant capacity but an increased penetration ability through the stratum corneum, reaching the viable epidermis and enhancing the stratum granulosum morphology. Both groups showed an increase in papillary depth, leading to improved dermis echogenicity and collagen fiber density. These findings contribute to the expanding understanding of nanotechnology-based skincare interventions for various skin conditions, highlighting potential avenues for future research and clinical applications for topical antioxidant therapy.

## 9. Future Perspectives

Diverse approaches have been developed to date for optimizing the benefits of antioxidant therapy, especially for topical applications on the skin. One promising approach is implementing nanotechnologies as drug delivery systems to improve the delivery of antioxidant agents to the skin, minimize drug irritation, and protect agents from environmental degradation. Various nanocarrier systems have been developed for topical antioxidant therapy, each with their respective advantages and limitations (Table 5). Selecting an appropriate nanocarrier is crucial for optimal therapy outcomes. When selecting nanocarrier systems for antioxidant agents, it is crucial to consider the physicochemical properties of the compounds. For instance, lipid-based nanocarriers are suitable for lipophilic drugs, while other systems can be used for both hydrophilic and lipophilic antioxidants. This will help ensure an optimal delivery and the effectiveness of the antioxidants. Due to differences in penetration ability among nanocarrier systems, the target delivery should also be initially appointed.

In addition to the advantages offered by nanotechnology, the safety issue of applying this technology should be carefully evaluated. Due to their unique characteristics, nanoparticles can penetrate deeper into the skin and spread uncontrollably throughout the body, leading to various adverse effects which highly depend on the accumulation location and concentration. These adverse effects occur when compounds bind to unintended targets, form reactive metabolites, or interact nonspecifically with biological macromolecules [185]. When it comes to delivering antioxidants, off-target effects can disrupt the cellular redox homeostasis, potentially leading to a state known as reductive stress, which is the opposite of oxidative stress [186]. Depending on where it occurs, reductive stress can have harmful effects on cells. For instance, chronic reductive stress has been associated with various pathological conditions, including heart failure, impaired cell growth responses, reduced mitochondrial function, and decreased cellular metabolism [187,188]. Moreover, these off-target interactions can significantly undermine the efficacy of antioxidant treatments on the skin. One effective strategy to mitigate this issue is to control the particle size of the nanocarriers. Additionally, incorporating targeting ligands can enhance the specific delivery of antioxidants to their intended site of action on the skin.

Moreover, induction of an immune response has been identified as a common occurrence during nanotechnology-based therapies. Therefore, the development of nanotechnology-based antioxidant therapies should not only aim for beneficial effects but should also include an analysis of the safety aspects. It is also essential to prioritize the use of materials that are both biocompatible and biodegradable. Moreover, minimizing toxic organic solvents during nanoparticle synthesis is crucial to ensure product safety. When applying toxic organic solvents is unavoidable, ensuring that the solvent residue on the final product is within its safety level is crucial.

In recent decades, nanotechnology has evolved as a powerful drug delivery system due to its unique characteristics and ability to target specific organs, tissues, cells, and even organelles inside the cells. This ability can be exploited for topical antioxidant therapy to improve the therapeutic benefits for the patients. By introducing a specific ligand on the surface of nanoparticles, we can control the biodistribution of the nanoparticles, making it possible to target a specific region of the body. The elucidation of specific receptors in the skin presents a noteworthy avenue for future research. Furthermore, the strategic targeting of particular organelles within skin cells may constitute a promising approach for the advancement of topical antioxidant therapies. As the powerhouse of the cells and the primary source of ROS, mitochondria can be a potential target for antioxidant therapy. Therefore, the specific targeting of mitochondria in the skin cells may become a challenging and innovative approach for topical antioxidant therapy.

It is essential to comprehend the current challenges, particularly in clinical trials and real-world applications, to advance effectively. Previous studies have demonstrated that nanocarriers outperform conventional formulations in terms of penetration, targeting, and controlled release. However, several constraints including stability under physiological conditions, regulatory hurdles, and scalability of production hinder the advancement of nanoformulations [14,189,190]. The absence of extensive clinical trials and real-world application data, particularly observational studies, for chronic dermatological conditions like melasma and dermatitis constitutes a notable obstacle [13], especially concerning antioxidants derived from natural resources. As of December 2024, a total of 258 studies have been conducted on the topics of antioxidants and skin conditions, with 54 of these studies currently active [191]. However, only one study addresses nanoformulations [192], confirming the previously mentioned challenges.

## 10. Conclusions

In summary, this review highlights the importance of topical antioxidant therapy in combating oxidative damage and the innovative role of nanotechnology in enhancing the delivery and effectiveness of antioxidants, including natural-resource-derived antioxidants, through various nanocarrier systems such as nanoemulsions, liposomes, SLNs, NLCs, and polymeric nanoparticles. These nanocarriers are vital to improve stability, drug release, penetration, and targeted delivery of antioxidants, ensuring better skin health outcomes. The limitations in this area of research include inconsistent and discontinuous results between in vitro and clinical studies, insufficient clinical trial evidence supporting effectiveness, and technological challenges related to large-scale production processes and the stability of nanocarriers. Furthermore, enhancing our understanding of the specific interactions among nanoparticles, antioxidant molecules, and biological targets is crucial, as is navigating the regulatory landscape for implementing this technology. The review also points out the potential of natural resources as a source of antioxidant agents, as well as the current status of the clinical application of antioxidant therapy. To advance the research in this field, carefully considering the safety and biocompatibility of nanocarriers under development is crucial. Additionally, exploring targeted delivery strategies, such as receptor-specific drug delivery and mitochondria-targeted antioxidant therapy, are essential to maximize the benefits of topical antioxidant treatments for skin protection and overall health maintenance.

## Figures and Tables

**Table 1:** List of natural resources with a potential antioxidant activity.

Name of Plant	Extraction Solvent	Extraction Method	Identified Responsible Compounds	Refs.
*Phyllanthus emblica*	Water	Infusion	Ascorbic acid	[68]
*Rosa damascene*	Water	Infusion	Polyphenols	[68]
*Stevia rebaudiana*	Water	Infusion	Phenolics and flavonoids	[68]
*Paeonia officinalis*	Methanol	Accelerated solvent extraction	Gallic acid derivatives	[69]
*Salvia nemorosa*	Methanol/water (80%, *v*/*v*)	Ultrasound-assisted extraction	Rosmarinic acid	[70]
*Salvia macrochlamys*	Methanol/water (80%, *v*/*v*)	Ultrasound-assisted extraction	Rosmarinic acid	[70]
Homnin black rice and Munpu red rice	Acidified ethanol	Microwave-assisted extraction	Phenolic, anthocyanins, and proanthocyanidins	[71]
*Cinnamomum* sp.	n.a.	n.a.	Eugenol	[72]
*Syzygium aromaticum*	n.a.	n.a.	Eugenol	[72]
*Centella asiatica*	50% ethanol	Sonication	Kaempferol, quercetin, rutin	[46]

n.a. = not available.

**Table 3:** Summary of antioxidant delivery systems to the skin using the liposome technology.

Antioxidant	Liposome Composition	Preparation Method	Outcomes	Refs.
Vitamin C	Soybean lecithin and sodium cholate	Not defined	Improves skin penetration and the photoprotective effects of vitamin C	[124]
Antioxidant complex	Phosphatidylcholine	Thin-film hydration	Restores pro-inflammatory cytokines of photoaging skin	[125]
Astaxanthin	Soy lecithin and cholesterol	Ethanol injection	Improves the stability of astaxanthin during storage	[126]
Astaxanthin	Soy lecithin and cholesterol, coated with chitosan	Thin-film hydration coupled with high-pressure homogenization	Improves the stability of astaxanthin and its antioxidant activity	[127]
Caffeic acid	Egg phosphatidylcholine and cholesterol	Reverse-phase evaporation	Enhances skin penetration by caffeic acid and retain its antioxidant activity	[128]
CoQ_10_	Phospholipid-Lipoid S100 and cholesterol	Thin-film hydration	Enhances cell proliferation under stress oxidative conditions	[129]
Retinyl palmitate	l-α-phosphatidylcholine	Thin-film hydration	Prolongs skin retention and provides a skin hydration effect	[107]

**Table 4:** Summary of antioxidant delivery using SLNs.

Antioxidant	Lipid	Surfactant	Result	Refs.
Retinyl palmitate	Compritol^®^ 888 ATO	Sodium lauryl sulphate and Span^®^ 80	Improves photostability and is dominantly retained in the superficial skin layer	[107]
Resveratrol	Compritol^®^ 888 ATO	Poloxamer 188 and Tween^®^ 80	Dominantly accumulated in the epidermis	[140]
Alpha-lipoic acid	Apifil	Pluronic^®^ F68	Sustained drug release profile	[106]
Lutein	Carnauba wax	Plantacare^®^ 810	Strong drug photoprotective effect	[141]
Tretinoin	Glyceryl monostearate	Epikuron 200	Improves tretinoin photostability and reduces skin irritation	[142]
Retinyl palmitate	Glyceryl palmitostearate	PEG-32 glyceryl stearate	Improves skin delivery by a factor of 4.8	[143]
Idebenone	Cetyl palmitate	Isoceteth and glyceryl oleate	Most of the drugs remain accumulated in the upper skin layers	[144]
Idebenone (pyroglutamic acid ester)	Cetyl palmitate	Glyceryl oleate	Improves skin hydration effects	[145]

**Table 5:** The advantages and limitations of nanocarrier systems as topical antioxidant delivery systems.

Nanocarrier Type	Advantages	Limitations
Nanoemulsions	○ Provide protection to the antioxidants ○ The particle size can be engineered ○ Drug release is relatively faster compared to other lipid-based nanocarriers	○ The presence of surfactants may be a potential cause of toxicity and irritation ○ Nanoemulsions are thermodynamically unstable systems
Liposomes	○ Potential as a carrier for both hydrophilic and hydrophobic antioxidants ○ Can be modified with targeting ligands and other functional molecules ○ Provide prolonged skin retention depending on the characteristics of the phospholipid	○ The residue of organic solvents in the synthesis process can be a potential source of toxicity and irritation ○ Mass production is still a challenge in liposome technology
SLNs	○ Potential candidates for delivering hydrophobic antioxidants ○ Preserve the antioxidant activity ○ Provide occlusive effects on the skin ○ Can be used to control the drug release for long periods of time relative to other lipid-based nanocarriers	○ The incorporation of hydrophilic antioxidants is possible but challenging ○ Relatively low encapsulation efficiency compared to NLCs ○ Drug expulsion may occur during storage
NLCs	○ Suitable for delivering hydrophobic antioxidants ○ Moderate drug release rate compared to other lipid-based nanocarriers ○ Protect antioxidants from degradation ○ Minimum drug leakage during storage	○ Incorporating hydrophilic antioxidants is possible, but it presents certain challenges ○ The presence of surfactants may cause irritation and sensitization
Polymeric nanoparticles	○ Suitable for delivering both hydrophilic and hydrophobic antioxidants ○ Protect antioxidants from degradation ○ Drug release rate can be engineered	○ Premature drug release ○ Subject to particle aggregation ○ Safety issue related to the main building block of the polymer

## List of Abbreviations

4nBR	4-n-butylresorcinol
ADP	Adenosine diphosphate
AGA	Androgenic alopecia
ATP	Adenosine triphosphate
BHA	Butylated hydroxy anisole
BHT	Butylated hydroxy toluene
CAT	Catalase
CO	Clove essential oil
CoQ_10_	Coenzyme Q_10_
DCP	Dicetyl phosphate
DNA	Deoxyribonucleic acid
DPPH	2,2-diphenyl-1-picrylhydrazyl
DSBs	DNA strand breaks
ETC	Electron transport chain
GPx	Glutathione peroxidase
GR	Glutathione reductase
GRAS	Generally recognized as safe
GSH	Glutathione
GSH-px	Glutathione peroxidase
MASI	Melasma area and severity index
MDA	Malondialdehyde
MMP-9	Matrix metalloproteinase 9
NLC	Nanostructured lipid carrier
NOXs	NADPH oxidases
O/W NE	Oil in water nanoemulsion
PAHs	Polycyclic aromatic hydrocarbons
PCL	Polycaprolactone
PEG	Polyethylene glycol
PM	Particulate matters
Prxs	Peroxiredoxins
RNS	Reactive nitrogen species
ROS	Reactive oxygen species
RSV	Resveratrol
SLN	Solid lipid nanoparticle
SOD	Superoxide dismutase
TA	Tranexamic acid
UV	Ultraviolet
UVA	Ultraviolet A
UVB	Ultraviolet B
VCO	Virgin coconut oil
VOCs	Volatile organic compounds
W/O NE	Water in oil nanoemulsion
XO	Xanthine oxidase
